# On Energy–Information Balance in Automatic Control Systems Revisited

**DOI:** 10.3390/e22111300

**Published:** 2020-11-15

**Authors:** Vladimir Rubtsov

**Affiliations:** 1 Département de Mathématiques, LAREMA UMR 6093 du CNRS, Université d’Angers, 49045 Angers, France; volodya@univ-angers.fr; 2 Theory Division, Institute for Theoretical and Experimental Physics, 117218 Moscow, Russia; 3 Institute of Geometry And Physics, 34151 Trieste, Italy

**Keywords:** Pinsker information rate, entropy rate, Legendre duality

## Abstract

We revise and slightly generalize some variational problems related to the “informational approach” in the classical optimization problem for automatic control systems which was popular from 1970–1990. We find extremals for various degenerated (derivative independent) functionals and propose some interpretations of obtained minimax relations. The main example of such functionals is given by the Gelfand–Pinsker–Yaglom formula for the information quantity contained in one random process in another one. We find some balance relations in a linear stationary one-dimensional system with Gaussian signal and interpret them in terms of Legendre duality.


*To Nikolay Petrovich Bukanov with my gratitude and admiration*


## 1. Introduction

An informational approach to an opimality criteria for a control system synthesis was proposed by Bukanov in [1]. He has considered the following one-dimensional, control system.

**Remark** **1.** *The “subtraction” and “addition” notation of the diagram nodes means that f.e. the input function X(t)=U(t)−P(t) and $Z\left(t\right)=Y\left(t\right)-Y_{0}\left(t\right)$ while V(t)=Z(t)+N(t).*

His choice of an informational criterion was motivated by a straightforward engineering application: studies of the automatic board control system of the passenger jet TU-154. Mathematically, he had used the classical Gelfand–Pinsker–Yaglom functional formula [2]

$$J_{U,Z}=-\frac{1}{4\pi }\int_{-\infty }^{+\infty }\log\left(1-R_{UZ}^{2}\right)d\omega$$

for the amount of information about the one random process (the defect or the error signal *Z*) contained in another such process (the control signal *U*), where $R_{UZ}$ denotes the mutual correlation function for these processes.

Pinsker had re-written this functional in the following form:

(1) $$J_{U,Z}=\frac{1}{4\pi }\int_{-\infty }^{+\infty }\log\frac{s_{U}s_{Z}}{s_{U}s_{Z}-{\left|s_{UZ}\right|}^{2}}d\omega .$$

Here, $S_{U},S_{Z}$ are spectral densities of the powers of the control signal *U* and the defect signal *Z*, $S_{UZ}$ -is the mutual spectral density.

Bukanov has expressed the functional variables of (1) in terms of the object-regulator transfer functions and spectral densities of the perturbations and has obtained the following explicit integral:

(2) $$J_{U,Z}=\frac{1}{4\pi }\int_{-\infty }^{+\infty }\log\frac{{\left(R_{0}s_{P}\right)}^{2}+{\left(R_{r}s_{N}\right)}^{2}+\left(1+R_{0}^{2}R_{r}^{2}\right)s_{N}s_{P}}{\left(1+R_{0}^{2}R_{r}^{2}+2R_{0}R_{r}\cos\left(\phi_{0}+\phi_{r}\right)\right)s_{N}s_{P}}d\omega .$$

The transfer functions of the object and the regulator are represented by the frequency responses $K_{0}\left(j\omega \right)=R_{0}\exp\left(j\omega \right)$ and $K_{r}\left(j\omega \right)=R_{r}\exp\left(j\omega \right).$ (We shall describe below the detailed reduction of the Pinsker Formula (1) to the Bukanov’s expression (2) in a slightly general context.)

**Figure 1:**
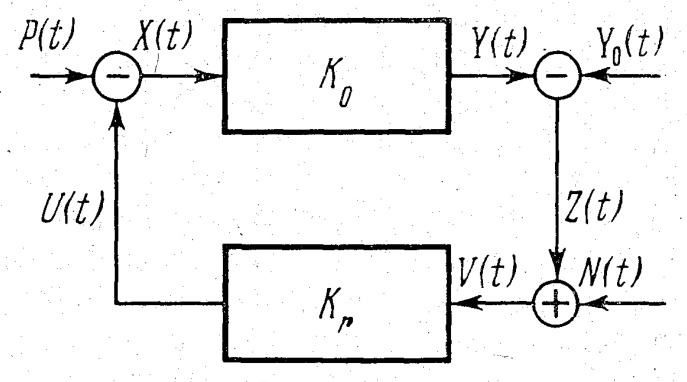
This is a figure from [1]. Here, $K_{0}$ denotes the operator object which transform the input function X(t) to the output function Y(t) which should be compared with the given function $Y_{0}$ (the program function). The “defect” signal *Z* together with a measurement “noise” *N* forms the input of a regulator given by an operator $K_{r}$. The resulting control signal *U* with an external perturbation *P* goes back to the input of the object operator $K_{0}$.

The aim of this paper to revise and to generalize this optimal regulator problem (for a class of linear stationary systems with non-zero program ($Y_{0}$)) as the classical optimization problem for automatic control system of Figure 1. We get the following results:

- find extremals for a generalization of (1) and for some other degenerated (derivative independent) “informational” functionals (the main example of such functionals is given by the Pinsker formula for the entropy quantity of one random process in another.)
- obtained some minimax relations and propose its new interpretations as an “energy–balance equilibrium” in a spirit of the Brillouin’s and Schrodinger’s ideas of “negentropy” (we remind briefly this notion in the Discussion section).
- write our balance relations in a linear stationary one-dimensional system with Gaussian signals and interpret them in terms of Legendre duality.

We would like to stress and warn a mathematical purist reader that this manuscript is not a “mathematical paper” in a formal sense of this notion. We (almost) never discuss and almost never precise any “existence and finitude conditions”. We would rather prefer formal manipulations whenever they exist. Therefore, we decided not to overload these small notes with probability and measure theory precise statements, technicalities, and terminology. We refer all interested readers to the book [2] for all rigorous conditions and other pure mathematical details and statements.

### 1.1. The Origins

The famous Shannon’s formula [3] provides a facility to measure the capacity of a communication channel in a presence not only of *white noise* but also in the case when the transmission is perturbed by any Gaussian noise with the power spectrum function ϵ(ω) proportional to the square module of a filter transfer function y(ω):

$$\epsilon \left(\omega \right)={\kappa |y\left(\omega \right)|}^{2}.$$

We recall it because it is probably the first when implicitly the informational characteristics are stipulated by some “energy” constraint. Namely, let the power of the “transmitter” is bounded by some (positive) quantity $A^{2}$ such the spectral function of the transmitted signal is s(ω):

(3) $$\int_{\omega_{0}}^{\omega_{1}}s\left(\omega \right)d\omega =A^{2}.$$

The Shannon formula we have mentioned above for the total capacity of s(ω) reads for the frequency bandwidth $\left[\omega_{0},\omega_{1}\right]$ as

$$C_{s,\epsilon }=\int_{\omega_{0}}^{\omega_{1}}\log\left(1+\frac{s(\omega )}{\epsilon (\omega )}\right)d\omega .$$

Now, it is easy to give a mathematical answer to the natural question: what should we do to get a maximal transmission rate for the given constraint (3)? Shannon proposes to consider the variational problem with the Lagrange multiplier:

(4) $$\max\{\int_{\omega_{0}}^{\omega_{1}}\left[\log\left(1+\frac{s(\omega )}{\epsilon (\omega )}\right)+\lambda s\left(\omega \right)\right]d\omega \}$$

which gives the condition

$$\lambda +\frac{1}{s(\omega )+\epsilon (\omega )}=0$$

and implies that s(ω)+ϵ(ω) is a constant. Going further, we put $s\left(\omega \right)=-\frac{1}{\lambda }-\epsilon \left(\omega \right)$ in (3) and obtain

$$\lambda =-\frac{\omega_{1}-\omega_{0}}{A^{2}+B^{2}},$$

where $B^{2}=\int_{\omega_{0}}^{\omega_{1}}\epsilon \left(\omega \right)d\omega$ and the extremal $s_{max}\left(\omega \right)=\frac{A^{2}+B^{2}}{\omega_{1}-\omega_{0}}-\epsilon \left(\omega \right)$, while

$$C_{s,\epsilon }^{max}=\left(\omega_{1}-\omega_{0}\right)\log\frac{A^{2}+B^{2}}{\omega_{1}-\omega_{0}}-\int_{\omega_{0}}^{\omega_{1}}\epsilon \left(\omega \right))d\omega .$$

In this paper, we are going to apply these beautiful observations of the founding father of Theory of Information to some (degenerated) variational problems with power constraints arising in the optimal control of automatic systems.

### 1.2. Review of Some Previously Known Results

The informational criteria for a control system synthesis were very popular in applied automatic control and measure systems in 1970–1980. We should mention here relevant to our interests works of academician Petrov’s school researchers [4]. They were concentrated on the questions of correctness and regularization (in sense of Tikhonov) the control and statistical optimization problems. Their most important and most interesting (to our aims ) input has concluded in a study of a connection between the regularization and a correctness of problems in a one side and an approach motivated by the above Shannon channel capacity formulae in the other. Mathematically, their main tool was based on the Wiener filter theory and related integral equations of the Kolmogorov–Wiener type (see, for example, [5]).

It seems that the system considered by N. Bukanov was ideologically similar to models elaborated by Petrov’s school researchers [5,6]. In the same time, one should stress that Bukanov’s choice of the informational criterion has some advantages. Its mathematical toolbox does not appeal to integral equations and was strongly motivated as we have mentioned by engineering applications to numerical computations related with an optimization of the jet TU-154 automatic board control system.

We shall compare both approaches—[1,5,6] for some basic examples of control linear systems.

We remind that, in [1], a solution was proposed for the variational problem in the class of linear stationary systems with zero program ($Y_{0}=0$) and Gaussian perturbation signal functions. Namely, Formula (1) makes sense exactly in this class of random functions admissible in [2].

More precisely, the main result of [1] is that the (degenerated) variational problem for the functional (2) has the following extremals:

(5) $$R_{r}=R_{0}\frac{s_{P}}{s_{N}},\;\phi_{r}=-\phi_{0},$$

which are minimized at the same time as the functional of the error signal dispersion:

(6) $$D_{Z,Z}=\int_{-\infty }^{+\infty }\frac{R_{0}^{2}\left(s_{P}+R_{r}^{2}s_{N}\right)}{1+R_{0}^{2}R_{r}^{2}+2R_{0}R_{r}\cos\left(\phi_{0}+\phi_{r}\right)}d\omega$$

and the functional of the control signal power dispersion:

(7) $$D_{U,U}=\int_{-\infty }^{+\infty }\frac{R_{r}^{2}\left(s_{N}+R_{0}^{2}s_{P}\right)}{1+R_{0}^{2}R_{r}^{2}+2R_{0}R_{r}\cos\left(\phi_{0}+\phi_{r}\right)}d\omega .$$

One can conclude that the extremal relations (5) between the object–regulator characteristics guarantee that the optimal regulator provides the minimal power to the object of control. Another interesting observation made in [1] is that, in spite of the functional (2) minimal value ${\left(J_{U,Z}\right)}_{min}=0$, it does not mean that the regulator does not use the information at all—it means only that there is a certain “informational balance”:

(8) $$J_{U,Z}=\left[C_{0,P,N}-Q_{0}\right]+\left[C_{r,P,N}-Q_{r}\right]$$

between the channel capacity

$$C_{0,P,N}=\frac{1}{4\pi }\int_{-\infty }^{+\infty }\log\left(1+R_{0}^{2}\frac{s_{P}}{s_{N}}\right)d\omega .$$

of the object channel in the presence of perturbations *P* and *N*, the channel capacity

$$C_{r,P,N}=\frac{1}{4\pi }\int_{-\infty }^{+\infty }\log\left(1+R_{r}^{2}\frac{s_{N}}{s_{P}}\right)d\omega .$$

of the regulator channel in the presence of perturbations *P* and *N*, and the (half-)differences

$$Q_{0}=\frac{1}{2}\left(Q_{0}^{\prime }-Q_{0}\prime \prime \right),\;Q_{r}=\frac{1}{2}\left(Q_{r}^{\prime }-Q_{r}\prime \prime \right)$$

of the informational performances $Q_{0},Q_{r}$ of the output of the object and the regulator channels with open ($Q_{0}^{\prime },Q_{r}^{\prime }$) and closed ($Q_{0}\prime \prime ,Q_{r}\prime \prime$) loop–controllers:

$$Q_{0}^{\prime }=\frac{1}{4\pi }\int_{-\infty }^{+\infty }\log\left(R_{0}^{2}\frac{s_{P}}{s_{N}}\right)d\omega ,Q_{r}^{\prime }=\frac{1}{4\pi }\int_{-\infty }^{+\infty }\log\left(R_{r}^{2}\frac{s_{N}}{s_{P}}\right)d\omega ,$$

$$Q_{0}\prime \prime =\frac{1}{4\pi }\int_{-\infty }^{+\infty }\log(|W_{ZP}{|}^{2}\frac{s_{P}}{s_{N}})d\omega ,Q_{r}\prime \prime =\frac{1}{4\pi }\int_{-\infty }^{+\infty }\log(|W_{UN}{|}^{2}\frac{s_{N}}{s_{P}})d\omega .$$

Here, $W_{ZP}$ and $W_{UN}$ are corresponding complex frequency responses (see details in [1]).

### 1.3. Further Generalizations and the Subject of the Paper

Further studies of this type optimal linear control systems were concerned to a natural generalization for the systems with a non-trivial program ($Y_{0}\neq 0$ ). At the same time, it was an interesting and mathematically natural question to study similar (degenerated) variational problems for various informational functionals similar to (1) with a “power” constraint of type (3).

The first steps in this direction were done by the author (in a collaboration with N. Bukanov and V. Kublanov). We have reported our partial results in the paper [7] which was, in fact, a short conference proceedings announcement. No details of those results were ever published (to the best of my knowledge). It should be mentioned that some results and the intrinsic ideology—to consider an “entropy criterion” of optimal regulation proposed in our paper [7] were rediscovered in 1986 in the announcement [8].

One of the aims of this manuscript is to revise and to generalize the results reported in [7] and to consider them in a general context of variational problems with Legendre–Fenchel dual functionals.

The paper is organized as follows: Section 2 contains a reminder and a generalization of basic results from [1]. We introduce the notations and remind main definitions of all important notions, objects, and formulas used in the manuscript—among them are: the Pinsker functionals of entropy and informational rates of one stationary Gaussian signal with respect to another, signal spectral densities, and transfer functions.

First, we remind readers about (and generalize in the case of a non-trivial Gaussian stationary program signal) the variational problem for the Pinsker functional in the form proposed by Bukanov. We observe and discuss (Proposition 1) an important formula that represents the Pinsker informational rate functional associated with the linear system in Figure 1 in a Shannon-like channel capacity form.

Theorem 2 in Section 3 solves two degenerate variational problems for channel capacities functionals related with the program channel and with the error signal perturbation channel. We compare the expressions for both channel capacities with the Petrov–Uskov Formula (6) from [6]. Theorem 3 (essentially reported in [7]) gives the main balance relations for the system on Figure 1. We want to stress that the observed similarity of these relations with the famous Brilliouin and Schrodinger “negentropy” notion appeared due to my numerous discussions with Bukanov, who also indicated a close connection with the information gain, or the Kullback–Leibler divergence (see, for example [9], for a modern geometric approach to “informational thermodynamics”). In Section 4, we consider another informational functionals with or without necessary connections with optimization of linear automatic control systems. Our main observation here is Theorem 4 which remarks that the formula for the second Pinsker main functional—the entropy rate of a random process relatively to another random process—can be interpreted like a Legendre duality relation for the first Pinsker informational rate functional.

## 2. Optimization of the Linear Control System with a Non-Trivial Stationary Gaussian Program

The results of the section (slightly) generalize and extend the material of the paper [1] and give a new interpretation of it. It is worth noting that our notations are different from the original and our motivations and reasonings do not base on possible engineering applications and are purely theoretical.

### 2.1. Useful Formulas and Notations

We collect here (for the convenience of readers) all basic theoretical formulas which we shall use throughout the text. We clarify (once and forever in the paper) that all random variables and random processes (discrete or continuous) we shall understand in the same sense as in the Pinsker book [2], and we refer a “mathematically oriented” reader to this book for precise definitions, description, and properties.

#### 2.1.1. Formulae

In what follows, we shall use the following important formulas. Let ξ,η be two *random functions* (= stationary (generalized) Gaussian random processes).

- The Gelfand–Pinsker–Yaglom functional (Formula (2.58) from [10]) for the amount of information about a random function contained in another such function:
  $$J_{\xi ,\eta }=\frac{1}{4\pi }\int_{-\infty }^{+\infty }\log\frac{s_{\xi }s_{\eta }}{s_{\xi }s_{\eta }-{\left|s_{\xi \eta }\right|}^{2}}d\omega =-\frac{1}{4\pi }\int_{-\infty }^{+\infty }\log\left(1-R_{\xi \eta }^{2}\right)d\omega$$
  where $s_{\xi }\left(\omega \right)$ and $s_{\eta }\left(\omega \right)$ are respectively the spectral densities of ξ and η and $s_{\xi \eta }\left(\omega \right)$—the mutual spectral density of ξ and η. Here,
  $$R_{\xi \eta }^{2}:=\frac{|s_{\xi \eta }{|}^{2}}{s_{\xi }s_{\eta }}$$
  is the common correlation function for the processes $s_{\xi }$ and $s_{\eta }$ (Formula (10.21) from [2]).
- Gelfand–Pinsker–Yaglom functional for Gaussian ξ,η=ξ+ν such that ξ and ν are non-correlated (Formula (2.61) from [10]):
  $$J_{\xi ,\eta }=\frac{1}{4\pi }\int_{-\infty }^{+\infty }\log\left(1+\frac{s_{\nu }}{s_{\xi }}\right)d\omega$$
- The entropy rate of a random variable ξ with respect to η was defined by Pinsker [2]. For two one-dimensional Gaussian stationary (in the wide sense) stochastic processes ξ and η, the formula for the entropy rate (Formula (10.5.7) from [2]) reads
  (9) $$H_{\xi ,\eta }=\frac{1}{4\pi }\int_{-\infty }^{+\infty }\left(\frac{s_{\xi }}{s_{\eta }}-1-\log\frac{s_{\xi }}{s_{\eta }}\right)d\omega$$
- For Gaussian ξ,η=ξ+ν such that ξ and ν are non-correlated:
  $$H_{\xi ,\eta }=\frac{1}{4\pi }\int_{-\infty }^{+\infty }\left(\frac{s_{\nu }}{s_{\xi }}-\log\left(1+\frac{s_{\nu }}{s_{\xi }}\right)\right)d\omega$$

#### 2.1.2. Generalized Variational Problem

We suppose now that the stationary Gaussian program $Y_{0}\neq 0.$ Then, the following system of linear operator equations with respect to the variables *U* and *Z* in p−coordinates of the Laplace transform for our control system in Figure 1 takes place:

(10) $$\left\{\begin{array}{c} K_{r}\left(p\right)Z\left(p\right)-U\left(p\right)=K_{r}\left(p\right)N\left(p\right),\\ Z\left(p\right)+K_{0}\left(p\right)U\left(p\right)=K_{0}\left(p\right)P\left(p\right)-Y_{0}\left(p\right). \end{array}\right.$$

The main determinant of the system

$$\Delta =\;\left|\begin{array}{cc} K_{r} & -1\\ 1 & K_{0} \end{array}\right|\;=1+K_{0}K_{r}\neq 0.$$

We remind readers about frequency representations $K_{0}\left(j\omega \right):=R_{0}\left(\omega \right)\exp\left(j\phi_{0}\left(\omega \right)\right)$ and $K_{r}\left(j\omega \right):=R_{r}\left(\omega \right)\exp\left(j\phi_{r}\left(\omega \right)\right)$

#### 2.1.3. Notations

For the automatic control system in Figure 1, we introduce the following notations:

1. $$S_{0,P,N}:=R_{0}^{2}s_{P}+s_{N}$$
2. $$S_{r,N,P}:=R_{r}^{2}s_{N}+s_{P}$$
3. $$S_{0,r,\phi }:=\left(1+2R_{0}R_{r}\cos\left(\phi_{0}+\phi_{r}\right)+R_{0}^{2}R_{r}^{2}\right)s_{P}s_{N}$$

In these notations, the functional (2) $J_{\xi ,\eta }$ for the processes ξ=U and η=Z is written as

(11) $$J_{U,Z}=\frac{1}{4\pi }\int_{-\infty }^{+\infty }\log\frac{S_{0,P,N}S_{r,N,P}}{S_{0,r,\phi }}d\omega$$

Solving the system (10) and calculating the spectral density of signals U,Z and denoting the program signal $s_{Y_{0}}$, we obtain

(12) $$\left\{\begin{array}{c} s_{Z}\left(\omega \right)=|W_{ZY_{0}}{|}^{2}\left(j\omega \right)s_{Y_{0}}\left(\omega \right)+|W_{ZN}{|}^{2}\left(j\omega \right)s_{N}\left(\omega \right)+{\left|W_{ZP}\right|}^{2}\left(j\omega \right)s_{P}\left(\omega \right),\\ s_{U}\left(\omega \right)=|W_{UY_{0}}{|}^{2}\left(j\omega \right)s_{Y_{0}}\left(\omega \right)+|W_{UN}{|}^{2}\left(j\omega \right)s_{N}\left(\omega \right)+{\left|W_{UP}\right|}^{2}\left(j\omega \right)s_{P}\left(\omega \right). \end{array}\right.$$

One can find the explicit expressions for the transfer functions W(jω) in Appendix B. We have computed the full functional generalizing functional (2):

(13) $$J_{U,Z}^{gen}=\frac{1}{4\pi }\int_{-\infty }^{+\infty }\log\frac{s_{P}\left[\left(s_{Y_{0}}+R_{0}^{2}S_{r,N,P}\right)\left(s_{Y_{0}}+S_{0,P,N}\right)\right]}{S_{0,r,\phi }\left(s_{Y_{0}}+R_{0}^{2}s_{P}\right)}d\omega$$

We see that $Y_{0}=0$ (13) and (11) coincide.

**Theorem** **1.** *The functional $J_{U,Z}^{gen}$ has the following extremals:*

(14) $$\left\{\begin{array}{c} \phi_{r}=\hat{\phi_{r}}:=-\phi_{0},\\ R_{r}=\hat{R_{r}}:=R_{0}\frac{s_{P}}{s_{N}}\left[1+\frac{s_{Y_{0}}}{R_{0}^{2}s_{p}}\right] \end{array}\right.$$

**Proof** **of Theorem 1.** We denote by L the “Lagrangian” density for the functional (13):

$$L\left(\phi_{r}\left(\omega \right),R_{r}\left(\omega \right)\right)=\log\frac{s_{P}\left[\left(s_{Y_{0}}+R_{0}^{2}S_{r,N,P}\right)\left(s_{Y_{0}}+S_{0,P,N}\right)\right]}{S_{0,r,\phi }\left(s_{Y_{0}}+R_{0}^{2}s_{P}\right)}$$

The “degeneracy” of (13) means that the Lagrangian density does not depend on derivatives of $\phi_{r}$ and $R_{r}$ and the Euler–Lagrange equations are replaced by easy and straightforward computations from the system of variations

(15) $$\left\{\begin{array}{c} \frac{\delta J_{U,Z}^{gen}}{\delta \phi_{r}}=0,\\ \frac{\delta J_{U,Z}^{gen}}{\delta R_{r}}=0 \end{array}\right.$$

(due to the basic lemma of the calculus of variations) by the usual function minimax conditions. The first variation gives

$$\frac{\delta J_{U,Z}^{gen}}{\delta \phi_{r}}=0\Rightarrow -\frac{2R_{0}R_{r}\sin\left(\phi_{0}+\phi_{r}\right)}{S_{0,r,\phi }\left(R_{0}^{2}s_{P}+s_{Y_{0}}\right)}=0$$

and we obtain the first condition in Theorem 1. The second condition is verifying with a bit more cumbersome computation and we put it in Appendix A. □

**Corollary** **1.**

1. *Taking $Y_{0}=0$ immediately obtains the extremals (5) of the functional (2);*
2. *The extremal minimal value of the functional (13) is*
  $$\hat{J_{U,Z}^{gen}}=0,$$
*but, exactly like in the case of the zero program (see (15) in [1]), it does not mean that the regulator of the control system does not use the information. It just means that there is a balance of various channel capacities generalizing (8). We shall discuss and interpret these generalizations in the next sections.*
3. *The same extremals (14) minimize the (generalized) functional of “power” error signal:*
  (16) $$D_{Z,Z}^{gen}=\int_{-\infty }^{+\infty }\frac{s_{P}s_{N}\left(s_{Y_{0}}+R_{0}^{2}S_{r,N,P}\right)}{S_{0.r,\phi }}d\omega .$$
4. *This extremal value easily computed*
  $$\hat{D_{Z,Z}^{gen}}={\left(\sigma_{Z}^{2}\right)}_{min}=\int_{-\infty }^{+\infty }\frac{s_{N}\left(s_{Y_{0}}+R_{0}^{2}s_{P}\right)}{s_{Y_{0}}+S_{0,P,N}}d\omega .$$
*and coincides with the minimal value of $D_{Z,Z}$ (14.1) in [1] for zero–program case.*
5. *The same is true for the similar generalization of the functional (7) for the power of the regulator. We omit the corresponding evident formulas generalizing (14.2) in [1].*

We shall obtain another remarkable representation for the functional (13) using the spectral densities of signals *Z* and $\hat{Z}$:

**Proposition** **1.** (17) $$J_{U,Z}^{gen}=\frac{1}{4\pi }\int_{-\infty }^{+\infty }\log\frac{s_{Z}}{s_{\hat{Z}}}d\omega$$

**Proof.**  We shall compute the spectral density $s_{Z}$ from the first equation of (12):

$$s_{Z}\left(\omega \right)=\frac{\left(s_{Y_{0}}+R_{0}^{2}S_{r,N,P}\right)s_{P}s_{N}}{S_{0,r,\phi }},$$

the spectral density $s_{\hat{Z}}$ putting the extremals (14) in the above formula and substituting both spectral densities in (17). □

This formula will be very useful in further interpretations in Section 4.

## 3. Capacity of Channels and Balance Theorems for the Linear Stationary Control System

Now, using the generalization for $Y_{0}\neq 0$, we can also generalize and extend the relations which we have reported in [7]. In the same time, we shall propose some new interpretations of these relations. We shall assume throughout this section that all signals are stationary and Gaussian.

### 3.1. Capacity of Channels via Differential Entropy

We recall that, for the capacity of the channel for the linear control system in Figure 1, the given program $Y_{0}$ can be computed via differential entropies (“reduced to a degree of freedom”) of this program and the error (defect) *Z* (see, for example, (ch. IV (12.5) in [11]):

(18) $$C_{Y_{0}}=H\left(Y_{0}\right)-H\left(Z\right)$$

where

(19) $$\left\{\begin{array}{c} H\left(Y_{0}\right)=\frac{1}{4\pi }\int_{-\infty }^{+\infty }\log\left(2\pi e\right)s_{Y_{0}}\left(\omega \right)d\omega ,\\ H\left(Z\right)=\frac{1}{4\pi }\int_{-\infty }^{+\infty }\log\left(2\pi e\right)s_{Z}\left(\omega \right)d\omega \end{array}\right.$$

and the expression (18) is written as

(20) $$C_{Y_{0}}=\frac{1}{4\pi }\int_{-\infty }^{+\infty }\log\frac{s_{Y_{0}}\left(\omega \right)}{s_{Z}\left(\omega \right)}d\omega .$$

Analogously, one can obtain the capacity of the channel for the linear control system relatively the perturbations *P* and *N*:

(21) $$C_{P,N}=H\left(Z\right)-H\left(P,N\right)$$

and

$$H\left(P,N\right)=\frac{1}{4\pi }\int_{-\infty }^{+\infty }\log\left(2\pi e\right)S_{0,P,N}\left(\omega \right)d\omega$$

such that

(22) $$C_{P,N}=\frac{1}{4\pi }\int_{-\infty }^{+\infty }\log\frac{s_{Z}\left(\omega \right)}{S_{0,P,N}\left(\omega \right)}d\omega .$$

**Remark** **2.** *It would be interesting to compare Formulas (20) and (22) with Formula (16) from [6]:*

(23) $$C=\int_{W}\log\frac{s_{m}\left(\omega \right)}{s_{\epsilon }\left(\omega \right)}d\omega .$$

**Figure 2:**
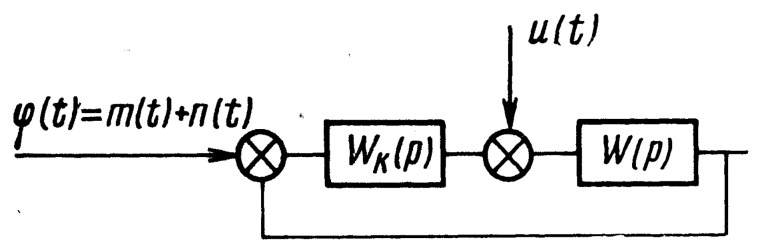
This is a figure from [5]. Here, W(p) denotes the transfer function of the operator object. The signal m(t) with the perturbation signal n(t) form a function ϕ(t) on the input of a regulator given by an operator $W_{k}\left(p\right)$. The system should reproduce the “useful” signal m(t) as an output.

*for a channel capacity of the linear system in Figure 2.*

**Remark** **3.** *Here the “cross” notation of the diagram nodes means the same as the “addition” nodes notation in Figure 1.*

Here, $s_{m}\left(\omega \right)$ and $s_{\epsilon }$ are spectral densities of the “useful” signal m(t) and the “error” signal ϵ(t) correspondingly.

The integral should be calculated over the total frequency band *W*. In particular, if u(t)=0 and *m* and *n* are non-correlated, then

(24) $$C=\int_{W}\log\frac{s_{m}\left(\omega \right)+s_{n}\left(\omega \right)}{s_{n}\left(\omega \right)}d\omega .$$

We assume that the perturbation n(t) has the spectral density with an additional “white noise” spectral density: $s_{n}\left(\omega \right)={\tilde{s}}_{n}\left(\omega \right)+c^{2}.$ We have from (24)

(25) $$C=\int_{W}\log\frac{s_{m}\left(\omega \right)+{\tilde{s}}_{n}\left(\omega \right)+c^{2}}{{\tilde{s}}_{n}\left(\omega \right)+c^{2}}d\omega .$$

The analog of the the balance relations (18) and (21) reads

$$H\left(n,m\right)-H\left(n,\epsilon \right)=C=\frac{1}{W}\int_{W}\log\frac{s_{m}\left(\omega \right)+{\tilde{s}}_{n}\left(\omega \right)+c^{2}}{{\tilde{s}}_{n}\left(\omega \right)+c^{2}}d\omega .$$

The authors of [5] argue that the entropy quantity H(n,ϵ) is a characteristic of information lost while the signal goes through the system of Figure 2, and the minimum of this loss is achieved when c≡0. When c→∞, the entropy H(n,ϵ)→H(n,m) i.e., the “error” entropy tends to the entropy of the “useful” signal and the channel capacity C goes to 0.

**Remark** **4.** *We should stress here that:*

1. *all signals are supposed to be stationary random Gaussian with zero expectation value;*
2. *the “optimality” solution in [5] was founded with the type “energy” constraint (3), which means in the context the minimality condition of the second momentum (“dispersion”) for the error signal $s_{\epsilon }:$*
  $$\int_{W}s_{\epsilon }d\omega =\int_{W}\frac{s_{m}\left(\tilde{s_{n}}+c^{2}\right)}{s_{m}+\tilde{s_{n}}+c^{2}}d\omega =\sigma_{\epsilon }^{2}$$
3. *The minimal value $\min\sigma_{\epsilon }^{2}$ is achieved when c=0 and this condition shows that the “energy” and informational criteria give the same result (compare with the similar conclusion for the case of the system in Figure 1)*
4. *The independency condition (the signals m(t) and n(t) are non-correlated) can be released, and this leads to the following generalization of capacity channel formula (24):*
  (26) $$C=\int_{W}\log\frac{s_{m}\left(s_{m}+s_{n}\right)}{s_{m}s_{n}+{\left|s_{mn}\right|}^{2}}d\omega .$$

### 3.2. Variational Problems for the Channel Capacity Functionals

Now, we need to substitute the spectral densities of all signals in Formulas (20) and (22) and, as a result, obtain the following two channel capacity functionals:

(27) $$C_{Y_{0}}=\frac{1}{4\pi }\int_{-\infty }^{+\infty }\log\frac{s_{Y_{0}}S_{0,r,\phi }}{s_{P}s_{N}\left(s_{Y_{0}}+R_{0}^{2}S_{r,N,P}\right)}d\omega$$

and

(28) $$C_{P,N}=\frac{1}{4\pi }\int_{-\infty }^{+\infty }\log\frac{s_{P}s_{N}\left(s_{Y_{0}}+R_{0}^{2}S_{r,N,P}\right)}{S_{0,P,N}S_{0,r,\phi }}d\omega$$

**Remark** **5.** *It is evident that the functional (28) generalizes the similar thing for the $Y_{0}=0$ case*

(29) $$C_{P,N}{|}_{Y_{0}=0}=\frac{1}{4\pi }\int_{-\infty }^{+\infty }\log\frac{s_{P}s_{N}R_{0}^{2}S_{r,N,P}}{S_{0,P,N}S_{0,r,\phi }}d\omega$$

*whose extremum under conditions (5) are*

(30) $$C_{P,N}^{min}{|}_{Y_{0}=0}=\frac{1}{4\pi }\int_{-\infty }^{+\infty }\log\frac{s_{P}s_{N}R_{0}^{2}}{S_{0,P,N}^{2}}d\omega$$

**Remark** **6.** *(One can straightforwardly check that this extremal value is a sum of the informational performances $\left(Q{\prime \prime }_{0},Q{\prime \prime }_{r}\right)$ of the object and the regulator channels with closed loop–controllers outputs under the conditions (5):*

$$C_{P,N}^{min,0}{{|}_{Y_{0}=0}=\left(Q{\prime \prime }_{0}+Q{\prime \prime }_{r}\right)|}_{\phi =\phi_{0},R_{r}=R_{0}\frac{s_{P}}{S_{N}}}.$$

These remarks definitely indicate that there are more general information–performance balance relations and we start to discuss them.

**Theorem** **2.** *The functional (27) has the maximal value*

(31) $$C_{Y_{0}}^{max}=\frac{1}{4\pi }\int_{-\infty }^{+\infty }\log\frac{s_{Y_{0}}}{s_{N}}\left[1+\frac{s_{N}}{s_{Y_{0}}+R_{0}^{2}s_{P}}\right]d\omega$$

*and the functional (28) has the minimal value*

(32) $$C_{P,N}^{min}=\frac{1}{4\pi }\int_{-\infty }^{+\infty }\log\frac{\left(s_{Y_{0}}+R_{0}^{2}s_{P}\right)s_{N}}{\left(s_{Y_{0}}+S_{0,P,N}\right)S_{0,P,N}}d\omega$$

*for the same set of extremals (14):*

$$\left\{\begin{array}{c} \phi_{r}=\hat{\phi_{r}}:=-\phi_{0},\\ R_{r}=\hat{R_{r}}:=R_{0}\frac{s_{P}}{s_{N}}\left[1+\frac{s_{Y_{0}}}{R_{0}^{2}s_{p}}\right] \end{array}\right.$$

**Proof** **of Theorem 2.** Both functionals are degenerated in the same sense as above. Therefore, their extremals are straightforwardly reduced to their Lagrangian densities’ usual minimax computations. Similarly, to determine a character of obtained extremums, it is enough to verify the Hessian matrices definiteness for these densities instead of computations of the second variation $\delta^{2}\left(C_{Y_{0}}\right)$ and $\delta^{2}\left(C_{N,P}\right)$. We omit these routine but tedious computations which are absolutely similar to those in Theorem 1. □

### 3.3. Informational Balance in the Linear Control System

The following theorem was obtained in a collaboration with Bukanov and Kublanov and was announced in [7].

**Theorem** **3.** *There are two balance relations between the channel capacities:*

1. *For the channel capacity of the program $Y_{0}$ and the informational rate in the control signal U in the “defect" signal Z:*
  (33) $$C_{Y_{0}}^{max}-C_{Y_{0}}=J_{U,Z}^{gen}.$$
2. *For the channel capacity of perturbations P and N and the above informational rate:*
  (34) $$C_{P,N}-{C^{min}}_{P,N}=J_{U,Z}^{gen}.$$

**Proof.**  Both identities are easily checked by manipulations with definitions of the corresponding channel capacities and computed extrema. Let us verify the first one:

$$C_{Y_{0}}^{max}-C_{Y_{0}}=\frac{1}{4\pi }\int_{-\infty }^{+\infty }\log\frac{s_{Y_{0}}}{s_{N}}\left[1+\frac{s_{N}}{s_{Y_{0}}+R_{0}^{2}s_{P}\left(\right)}\right]d\omega -\frac{1}{4\pi }\int_{-\infty }^{+\infty }\log\frac{s_{Y_{0}}S_{0,r,\phi }}{s_{P}s_{N}\left(s_{Y_{0}}+R_{0}^{2}S_{r,N,P}\right)}d\omega$$

$$=\frac{1}{4\pi }\int_{-\infty }^{+\infty }\log\frac{s_{P}\left(s_{Y_{0}}+R_{0}^{2}s_{P}+s_{N}\right)\left(s_{Y_{0}}+R_{0}^{2}S_{r,N,P}\right)}{S_{0,r,\phi }\left(s_{Y_{0}}+R_{0}^{2}s_{P}\right)}d\omega =J_{U,Z}^{gen}.$$

 □

**Remark** **7.** *Using (20), one can recast Formula (17) re-writing the first balance relation (33) as*

$$J_{U,Z}^{gen}=\frac{1}{4\pi }\int_{-\infty }^{+\infty }\log\frac{s_{Y_{0}}\left(\omega \right)}{s_{\hat{Z}}\left(\omega \right)}d\omega -\frac{1}{4\pi }\int_{-\infty }^{+\infty }\log\frac{s_{Y_{0}}\left(\omega \right)}{s_{Z}\left(\omega \right)}d\omega =\frac{1}{4\pi }\int_{-\infty }^{+\infty }\log\frac{s_{Z}\left(\omega \right)}{s_{\hat{Z}}\left(\omega \right)}d\omega .$$

*On other hand, it means that*

$$J_{U,Z}^{gen}=\frac{1}{4\pi }\int_{-\infty }^{+\infty }\log\left(2\pi e\right)s_{Z}\left(\omega \right)d\omega -\frac{1}{4\pi }\int_{-\infty }^{+\infty }\log\left(2\pi e\right)s_{\hat{Z}\left(\omega \right)}d\omega =H\left(Z\right)-H\left(\hat{Z}\right)$$

*which shows a similarity with the Brillouin “negentropy" principle which says that the system loses information during a transition from a state with low entropy to a state with higher entropy. (We include a brief description of this notion in the Discussion section below).*

*Naively speaking, the entropy H(Z) of the error signal of the linear system with *any* regulator is bigger (“more chaos") than the error signal entropy $H(\hat{Z})$ for the case of the system with optimal regulator:*

$$H\left(\hat{Z}\right)=H\left(Z\right)-J_{U,Z}^{gen}\geq 0.$$

*Similarly, the same result can be obtained from the second balance relation*

$$C_{P,N}-C_{P,N}^{min}=H\left(Z\right)-H\left(P,N\right)-H\left(\hat{Z}\right)+H\left(P,N\right)=J_{U,Z}^{gen}.$$

*One can imagine here the informational rate $J_{U,Z}^{gen}$ as an “informational difference” between Z and $\hat{Z}$ or as a “Brillouin–Schrödinger negentropy” [12].*

**Remark** **8.** *We should admit that the first balance relation (33) does not exist in the absence of the program signal ($Y_{0}=0$), while the second is a generalization of the previously described informational balance relation (8): The LHS of the identity (34) under condition $Y_{0}=0$ is:*

$$\left(C_{P,N}-{C^{min}}_{P,N}\right){|}_{Y_{0}=0}=\frac{1}{4\pi }\int_{-\infty }^{+\infty }\log\frac{s_{P}s_{N}R_{0}^{2}S_{r,N,P}}{S_{0,P,N}S_{0,r,\phi }}d\omega -\frac{1}{4\pi }\int_{-\infty }^{+\infty }\log\frac{s_{P}s_{N}R_{0}^{2}}{S_{0,P,N}^{2}}d\omega$$

(35) $$\begin{array}{c} =\frac{1}{4\pi }\int_{-\infty }^{+\infty }\log\frac{S_{0,P,N}S_{r,N,P}}{S_{0,r,\phi }}d\omega =J_{UZ}=\left(J_{UZ}^{gen}\right){|}_{Y_{0}=0.} \end{array}$$

*which is exactly the RHS of (34) in the same condition.*

## 4. Entropy Rate Functional for the Linear Stationary Control System

Now, we shall discuss the entropy rate functional (9) for the pair of Gaussian stationary error signal (*Z*) and the error signal with optimal regulator ($\hat{Z}$) in the linear control system above:

(36) $$H_{Z,\hat{Z}}=\frac{1}{4\pi }\int_{-\infty }^{+\infty }\left(\frac{s_{Z}}{s_{\hat{Z}}}-1-\log\frac{s_{Z}}{s_{\hat{Z}}}\right)d\omega .$$

Introduce the function $F\left(X\right):=\log\left(X\right)-1+\frac{1}{X}$ of a positive argument X>0 and study its behavior.

### 4.1. Properties of the Function F(X) and Legendre–Fenchel Transformation

**Lemma** **1.** *The function F(X) has the following (almost) evident properties:*

1. *F(X)≥0 and F(1)=0.*
2. *Let $X=\frac{u}{v}$ such that u>0;v>0, then*
  $$\log\left(\frac{u}{v}\right)\geq \left(1-\frac{v}{u}\right);$$
3. *If u=u(ω) and v=v(ω) are integrable on (R,dμ(ω)) with some measure μ(ω) and*
  $$\int_{R,\mu }ud\mu \left(\omega \right)=\int_{R,\mu }vd\mu \left(\omega \right)$$
*then*
  $$\int_{R,\mu }u\log\left(\frac{u}{v}\right)d\mu \left(\omega \right)\geq 0;$$
4. Let $Y=\frac{1}{X}$ and $F\left(Y\right):=F\left(\frac{1}{X}\right)$; then, the function F(Y)=Y−1−log(Y) admits the Legendre–Fenchel transformation $F^{*}\left(p\right)=-\log\left(1-p\right).$

**Proof.** 

1. To prove the first positivity property of F(X), we split the half line {X≥0} in two subsets: {0≤X<1} and {X≥1}. Start with the second subset: for 1≤t≤X, we have $\frac{1}{t}\geq \frac{1}{X}$ and
  $$\log X=\int_{1}^{X}\frac{dt}{t}\geq \int_{1}^{X}\frac{dt}{X}=\frac{1}{X}\int_{1}^{X}dt=\frac{X-1}{X}=1-\frac{1}{X}.$$
  In the first subset, one has similarly $-\frac{1}{t}\geq -\frac{1}{X}$ if X≤t<1 and
  $$\log X=-\int_{X}^{1}\frac{dt}{t}\geq -\int_{X}^{1}\frac{dt}{X}=1-\frac{1}{X}.$$
2. Tautological corollary of the computations in (1).
3. Using the inequality (2), we obtain:
  $$\int_{R,\mu }u\log\left(\frac{u}{v}\right)d\mu \left(\omega \right)\geq \int_{R,\mu }u\left(1-\frac{v}{u}\right)d\mu \left(\omega \right)=0;$$
4. The Legendre–Fenchel transformation $F^{*}\left(p\right)$ of the function F(Y) exists because the function is smooth and convex for Y>0. The only critical point $Y_{crit}=1$ with the critical value $F(Y_{crit})=0$ is obtained from $1-\frac{1}{Y}=0$. By the definition (see, f.e. [13], we put $p:=1-\frac{1}{Y}$ and find $Y\left(p\right)=\frac{1}{1-p}$. Then, we find the Legendre-dual function from $\frac{d}{dp}\left(F^{*}\left(p\right)\right)=\frac{1}{1-p},\;F^{*}\left(p\right)=-\log\left(1-p\right).$ We shall check that it satisfies the duality relation
  $$F^{*}\left(p\right)=pY\left(p\right)-F\left(Y\left(p\right)\right):$$
  $$pY\left(p\right)-F\left(Y\left(p\right)\right)=p\frac{1}{1-p}-\left(\frac{1}{1-p}-1-\log\left(\frac{1}{1-p}\right)\right)=-\log\left(1-p\right)=F^{*}\left(p\right).$$

 □

We note the famous Fenchel–Young inequality which reads in this case as

$$F\left(Y\right)+F^{*}\left(p\right)\geq Yp$$

which gives the evident inequality

1+log[Y(1−p)]≤Y(1−p).

### 4.2. Legendre Transformation Analogy for the Control System Functionals

We can observe that the Pinsker entropy rate functional (9) can be written in the form

$$H_{\xi ,\eta }=\frac{1}{4\pi }\int_{-\infty }^{+\infty }\left(\frac{s_{\xi }}{s_{\eta }}-1-\log\frac{s_{\xi }}{s_{\eta }}\right)d\omega =\frac{1}{4\pi }\int_{-\infty }^{+\infty }F\left(Y\left(\omega \right)\right)d\omega ,$$

where $Y\left(\omega \right):=\frac{s_{\xi }\left(\omega \right)}{s_{\eta }\left(\omega \right)}.$ Then, the Legendre–Fenchel dual functional should be

$$\frac{1}{4\pi }\int_{-\infty }^{+\infty }F^{*}\left(p\left(\omega \right)\right)d\omega ,$$

with $p\left(\omega \right)=1-\frac{1}{Y(\omega }=1-\frac{s_{\eta }\left(\omega \right)}{s_{\xi }\left(\omega \right)}.$ Using the part 4 of the Lemma 1, one can compute

$$F^{*}\left(p\left(\omega \right)\right)=-\log\left(1-p\left(\omega \right)\right)=-\log\left(\frac{s_{\eta }\left(\omega \right)}{s_{\xi }\left(\omega \right)}\right)=\log\left(\frac{s_{\xi }\left(\omega \right)}{s_{\eta }\left(\omega \right)}\right)=\log Y\left(\omega \right).$$

Thus, we have obtained the following Legendre duality relation:

$$\frac{1}{4\pi }\int_{-\infty }^{+\infty }F\left(Y\left(\omega \right)\right)d\omega +\frac{1}{4\pi }\int_{-\infty }^{+\infty }F^{*}\left(p\left(\omega \right)\right)d\omega =\frac{1}{4\pi }\int_{-\infty }^{+\infty }p\left(\omega \right)Y\left(\omega \right)d\omega .$$

Coming back to the Pinsker entropy rate functional Formula (9), one observes that the duality implies

(37) $$H_{\xi ,\eta }+\frac{1}{4\pi }\int_{-\infty }^{+\infty }\log\left(\frac{s_{\xi }\left(\omega \right)}{s_{\eta }\left(\omega \right)}\right)d\omega =\frac{1}{4\pi }\int_{-\infty }^{+\infty }\left(\frac{s_{\xi }\left(\omega \right)}{s_{\eta }\left(\omega \right)}-1\right)d\omega .$$

Formula (37) expressed the duality relation between the entropy rate and the functional $\frac{1}{4\pi }\int_{-\infty }^{+\infty }\log\left(\frac{s_{\xi }\left(\omega \right)}{s_{\eta }\left(\omega \right)}\right)d\omega$ for any pair of stationary Gaussian signals. Let us consider the case of the Pinsker entropy rate for our control system (36), i.e., $s_{\xi }\left(\omega \right)=s_{Z}\left(\omega \right)$ and $s_{\eta }\left(\omega \right)=s_{\hat{Z}}\left(\omega \right)$. In this case, (37) and (36) give us the Legendre duality condition between the functionals:

(38) $$H_{Z,\hat{Z}}+\frac{1}{4\pi }\int_{-\infty }^{+\infty }\log\left(\frac{s_{Z}\left(\omega \right)}{s_{\hat{Z}}\left(\omega \right)}\right)d\omega =\frac{1}{4\pi }\int_{-\infty }^{+\infty }\left(\frac{s_{Z}\left(\omega \right)}{s_{\hat{Z}}\left(\omega \right)}-1\right)d\omega .$$

Summing up the discussion in Section 4.2, one can formulate the following theorem:

**Theorem** **4.** *The Pinsker functional of the entropy rate of the error signal in the stationary linear control system with respect to the error signal of this control system with the optimal by the informational criterion regulator and the informational rate functional for these signals are in Legendre duality:*

(39) $$H_{Z,\hat{Z}}+J_{U,Z}^{gen}=\frac{1}{4\pi }\int_{-\infty }^{+\infty }\left(\frac{s_{Z}\left(\omega \right)}{s_{\hat{Z}}\left(\omega \right)}-1\right)d\omega .$$

**Remark** **9.** *We can also check that the the duality relation (37) can be easily generalized to the multidimensional case for two n− dimensional stationary Gaussian processes ξ and η such that $\xi =\left(\xi_{1},\ldots ,\xi_{n}\right),\eta =\left(\eta_{1},\ldots ,\eta_{n}\right)$ and such that the pairs $\left(\xi_{j},\eta_{j}\right)$ are pair-wise uncorrelated. Then, one can define n− dimensional analogues of entropy rate and informational rate functionals:*

$$H_{\xi ,\eta }=\frac{1}{4\pi }\int_{-\infty }^{+\infty }\left[\left(\sum_{j,k=1}^{n}s_{\xi_{j}\xi_{k}}\left(\omega \right)s_{\eta_{j}\eta_{k}}^{-1}\left(\omega \right)\right)-n-\log\frac{\det∥s_{\xi_{j}\xi_{k}}\left(\omega \right)∥}{\det∥s_{\eta_{j}\eta_{k}}\left(\omega \right)∥}\right]d\omega$$

*which can be simplified (because of the mutual independence of the signals ξ and η ) to*

(40) $$H_{\xi ,\eta }=\frac{1}{4\pi }\int_{-\infty }^{+\infty }\sum_{j=1}^{n}\left(\frac{s_{\xi_{j}\xi_{j}}\left(\omega \right)}{s_{\eta_{j}\eta_{j}}\left(\omega \right)}-1-\log\frac{s_{\xi_{j}\xi_{j}}\left(\omega \right)}{s_{\eta_{j}\eta_{j}}\left(\omega \right)}\right)d\omega ,$$

*where $∥s_{\xi_{j}\xi_{k}}\left(\omega \right)∥$ (resp. $∥s_{\eta_{j}\eta_{k}}\left(\omega \right)∥$) is the mutual spectral densities matrix of signal-components of ξ (resp.η) and $s_{\eta_{j}\eta_{k}}^{-1}\left(\omega \right)$ denotes the (jk) entry of the matrix $∥s_{\eta_{j}\eta_{k}}\left(\omega \right)∥.$*

*We see that one can apply the Legendre duality Formula (37) to each summand of (40) and re-write it as*

(41) $$H_{\xi ,\eta }+L_{\xi ,\eta }=\frac{1}{4\pi }\int_{-\infty }^{+\infty }\sum_{j=1}^{n}\left(\frac{s_{\xi_{j}\xi_{j}}\left(\omega \right)}{s_{\eta_{j}\eta_{j}}\left(\omega \right)}-1\right)d\omega ,$$

*where the functional*

$$L_{\xi ,\eta }=\frac{1}{4\pi }\int_{-\infty }^{+\infty }\log\frac{\det∥s_{\xi_{j}\xi_{k}}\left(\omega \right)∥}{\det∥s_{\eta_{j}\eta_{k}}\left(\omega \right)∥}d\omega =\frac{1}{4\pi }\int_{-\infty }^{+\infty }\sum_{j=1}^{n}\log\frac{s_{\xi_{j}\xi_{j}}\left(\omega \right)}{s_{\eta_{j}\eta_{j}}\left(\omega \right)}d\omega$$

*plays the role of the Pinsker informational rate functional (see chapters 10 and 11 in [2]) and for n=1 coincides with $\frac{1}{4\pi }\int_{-\infty }^{+\infty }\log\left(\frac{s_{\xi }\left(\omega \right)}{s_{\eta }\left(\omega \right)}\right)d\omega .$*

We want to stress that our multidimensional considerations are purely formal. We do not discuss the conditions of existence and finiteness for all formulas above and refer to [2,14] for all necessary mathematical details. We also do not know if these multidimensional generalizations can immediately apply to an optimal automatic control system problem, but we hope that such functional might be useful in comparison with the method of multidimensional optimization where the minimum of the error expectation value square and maximum of the information mean value criteria were used [15].

## 5. Discussion

The relationship between entropy and information was first discovered in the seminal work of Szilard [16]. Later, in the works of Brillouin [17], the negentropy principle of information was formulated, generalizing the second law of thermodynamics. According to this principle, both entropy and information should be considered jointly and cannot be interpreted separately. Brillouin had shown [18] that the definition of “information” leads to a direct connection between information and the negative of entropy (here is the abbreviation “negentropy”). Every experiment consumes negentropy (increases entropy) and yields information. The negentropy principle of information is a generalization of Carnot’s principle in thermodynamics that Brillouin had formulated in the following form: the amount of information contained in a physical system must be considered as a negative term in the total entropy of this system. If a system is isolated, it fulfills Carnot’s principle in its generalized form. According to this principle, the total entropy of the system does not decrease. Our Theorem 4 is a good illustration of these energy-information principle.

The functional $L_{\xi ,\eta }:=\frac{1}{4\pi }\int_{-\infty }^{+\infty }\log\left(\frac{s_{\xi }\left(\omega \right)}{s_{\eta }\left(\omega \right)}\right)d\omega$ enters in the Legendre duality relation (37) with the entropy rate functional $H_{\xi ,\eta }$ and has explicit interpretation in terms of the informational rate $J_{Z,\hat{Z}}$ in the case of the control system. The Legendre balance relation predicts some thermodynamical allusions going back to Gibbs thermodynamical potential relation:

S(E)+F(β)=βE,

which relates the Helmholtz free energy *F* to the entropy *S*, total energy *E*, and $\beta =\frac{1}{T}$ is inverse to the temperature (the Boltzman constant is supposed to be 1 here). If we shall follow the initial idea of Bukanov about a “similarity” of the informational rate functional $L_{\xi ,\eta }$ with the Brillouin negentropy N(ρ) and the information gain or Kullback–Leibler divergence I(p,q)=∫ρlogρdq which is “similar (up to sign) to the differential entropy expression”:

$$N\left(\rho \right)=I\left(\xi \right)+I\left(p,q\right)=-\int \xi \log\xi dq+I\left(p,q\right)=\int \log\frac{\rho }{\xi }dq$$

for Gaussian ξ, which has the same mean value as ρ. (notations and the statement from p. 15 of [9]), then we should conclude that, in our case, the informational rate functional $J_{\xi ,\eta }$ is rather a Legendre dual to the entropy rate $H_{\xi ,\eta }$, though it plays a “role of Hamiltonian” while $H_{\xi ,\eta }$ figured here as a “Lagrangian”. Following the “mechanical and thermodynamical” allusions of [9], we could interpret the results of our Theorems 1 and 2 as a (specified) example of the “principle of minimal gain information” and the balance relations in Theorem 3 show the connection with Jaynes principle of maximum entropy. It would interesting to give further interpretations in this line and to understand what the role of the “partition function” Z is, which, in our context, should naively read

$$L_{\xi ,\eta }=\int \log\frac{s_{\xi }}{s_{\eta }}d\omega =-\int \log Zd\omega .$$

This question, together with more serious comprehension of appropriate Lagrangian or canonical coordinates in these application approaches, would open a door to powerful invariant methods in the theory of information control systems.

There are many other possible developments to study, but all of them look more traditional. We could almost straightforwardly generalize our results using slightly more complicated cases of the capacity channel formulas in comparison with the results of Petrov et al. for signals with correlations, multidimensional systems generalizing the system in Figure 2, etc. The “physical realization” conditions are also beyond the scope of this paper.

## Appendix A. Proof of the Theorem 1, Second Condition

$$\frac{\delta J_{U,Z}^{gen}}{\delta R_{r}}=\frac{1}{4\pi }\int_{-\infty }^{+\infty }\frac{\delta }{\delta R_{r}}\left[\log\left(s_{Y_{0}}+R_{0}^{2}s_{P}+R_{0}^{2}R_{r}^{2}s_{N}\right)-\log S_{0,r,\phi }\right]d\omega$$

$$=\frac{1}{4\pi }\int_{-\infty }^{+\infty }\left[\frac{s_{N}R_{r}R_{0}}{\left(s_{Y_{0}}+R_{0}^{2}s_{P}+R_{0}^{2}R_{r}^{2}s_{N}\right)}-\frac{\cos\left(\phi_{0}+\phi_{r}\right)+R_{0}R_{r}}{1+2R_{0}R_{r}\cos\left(\phi_{0}+\phi_{r}\right)+R_{0}^{2}R_{r}^{2}}\right]d\omega .$$

Using the first condition of (14), one can re-write it as

$$\frac{1}{4\pi }\int_{-\infty }^{+\infty }\left[\frac{s_{N}R_{r}R_{0}}{\left(s_{Y_{0}}+R_{0}^{2}s_{P}+R_{0}^{2}R_{r}^{2}s_{N}\right)}-\frac{1+R_{0}R_{r}}{1+2R_{0}R_{r}+R_{0}^{2}R_{r}^{2}}\right]d\omega =$$

$$\frac{1}{4\pi }\int_{-\infty }^{+\infty }\left[\frac{s_{N}R_{r}R_{0}}{\left(s_{Y_{0}}+R_{0}^{2}s_{P}+R_{0}^{2}R_{r}^{2}s_{N}\right)}-\frac{1}{1+R_{0}R_{r}}\right]d\omega =\frac{1}{4\pi }\int_{-\infty }^{+\infty }\left[\frac{s_{N}R_{r}R_{0}-\left(s_{Y_{0}}+R_{0}^{2}s_{P}\right)}{\left(s_{Y_{0}}+R_{0}^{2}s_{P}+R_{0}^{2}R_{r}^{2}s_{N}\right)\left(1+R_{0}R_{r}\right)}\right]d\omega .$$

Now, using the basic lemma of variational calculus, we obtain the second condition in (14):

$$s_{N}R_{r}R_{0}=s_{Y_{0}}+R_{0}^{2}s_{P}.$$

## Appendix B. Formulas for Transition Functions in Frequency Variables

We shall display for completeness reasons the formulas (from [1,7]) for transition functions of the linear system in Figure 1 which we have used in computations of the spectral densities $s_{U}$ and $s_{Z}$ (12):

$$\left\{\begin{array}{cc} W_{ZY_{0}}\left(j\omega \right)=\frac{-1}{1+R_{0}R_{r}}, & W_{ZN}\left(j\omega \right)=\frac{-R_{0}R_{r}}{1+R_{0}R_{r}}\\ W_{ZP}\left(j\omega \right)=\frac{R_{0}}{1+R_{0}R_{r}}, & W_{UY_{0}}\left(j\omega \right)=\frac{-R_{r}}{1+R_{0}R_{r}}\\ W_{UN}\left(j\omega \right)=\frac{R_{r}}{1+R_{0}R_{r}}, & W_{UP}\left(j\omega \right)=\frac{R_{0}R_{r}}{1+R_{0}R_{r}}. \end{array}\right.$$

## References

[1] Bukanov N.P. (1972). Information criterion of optimality of automatic control systems. Avtomat. Telemekh..

[2] Pinsker M.S. (1960). Information and Informational Stability of Random Variables and Processes.

[3] Shannon C. (1948). A Mathematical Theory of Communication. Bell Syst. Tech. J..

[4] Petrov B.N., Petrov V.V., Ulanov G.M., Zaporczets A.W., Uskov A.S., Kotchubievsky l.L. (1969). Stochastic Process in Control and Information Systems, Survey Paper 63.

[5] Petrov V.V., Uskov A.S. (1970). Informational aspects of the regularization problem. Doklady AN SSSR.

[6] Petrov V.V., Uskov A.S. (1970). Channel capacities of feed-back informational systems with internal perturbations. Doklady AN SSSR.

[7] Bukanov N.P., Kublanov V.S., Rubtsov V.N. About a question on a synthesis of an automatic control system optimal with respect to an informational criterion. Proceedings of the All-Union Conference Optimization and Efficiency of Systems and Processes in Civil Aviation, MIIGA.

[8] Petrov V.V., Sobolev V.I. (1986). Entropy approach to a quality criterial of automatic control systems. Doklady AN SSSR.

[9] Lychagin V.V., Kycia R.A., Ulan M., Schneider E. (2019). Contact Geometry, Measurement, and Thermodynamics. Nonlinear PDEs, Their Geometry, and Applications’.

[10] Gelfand I.M., Yaglom A.M. (1957). Computation of the amount of information about a stochastic function contained in another such function. Uspehi Mat. Nauk.

[11] Tarasenko F.P. (1963). Introduction in the course of Information Theory.

[12] Schrödinger E. (1947). What Does Life Is from the Physics Point of View?.

[13] Arnold V.I. (1989). Mathematical Methods of Classical Mechanics.

[14] Pinsker M.S. (1960). Entropy, entropy rate and entropy stability of Gaussian random variables and processes. Doklady AN SSSR.

[15] Petrov V.V., Zaporozhets A.V. (1975). Principle of developable systems. Doklady AN SSSR.

[16] Szilard L. (1929). On the reduction of entropy in a thermodynamic system by the intervention of intelligent beings. Z. Phys..

[17] Brillouin L. (1960). Science and Information Theory.

[18] Brillouin L. (1961). Thermodynamics, statistics, and information. Am. J. Phys..

